# Intestinal oxygen utilisation and cellular adaptation during intestinal ischaemia–reperfusion injury

**DOI:** 10.1002/ctm2.70136

**Published:** 2024-12-26

**Authors:** Paraschos Archontakis‐Barakakis, Theodoros Mavridis, David‐Dimitris Chlorogiannis, Georgios Barakakis, Eleni Laou, Daniel I. Sessler, George Gkiokas, Athanasios Chalkias

**Affiliations:** ^1^ Department of Medicine Redington‐Fairview General Hospital Skowhegan Maine USA; ^2^ Department of Neurology Tallaght University Hospital (TUH)/The Adelaide and Meath Hospital incorporating the National Children's Hospital (AMNCH) Dublin Ireland; ^3^ Department of Radiology Brigham and Women's Hospital, Harvard Medical School Boston Massachusetts USA; ^4^ Faculty of Health Sciences School of Medicine Aristotle University of Thessaloniki Thessaloniki Greece; ^5^ Department of Anesthesiology Agia Sophia Children's Hospital Athens Greece; ^6^ Center for Outcomes Research and Department of Anesthesiology UTHealth Houston Texas USA; ^7^ Outcomes Research Consortium^®^ Houston Texas USA; ^8^ Second Department of Surgery Aretaieion University Hospital, National and Kapodistrian University of Athens Athens Greece; ^9^ Institute for Translational Medicine and Therapeutics University of Pennsylvania Perelman School of Medicine Philadelphia Pennsylvania USA; ^10^ Department of Critical Care Medicine Tzaneio General Hospital Piraeus Greece

**Keywords:** dysoxia, hypoxia, intestine, ischaemia–reperfusion injury, oxygen

## Abstract

The gastrointestinal tract can be deranged by ailments including sepsis, trauma and haemorrhage. Ischaemic injury provokes a common constellation of microscopic and macroscopic changes that, together with the paradoxical exacerbation of cellular dysfunction and death following restoration of blood flow, are collectively known as ischaemia–reperfusion injury (IRI). Although much of the gastrointestinal tract is normally hypoxemic, intestinal IRI results when there is inadequate oxygen availability due to poor supply (pathological hypoxia) or abnormal tissue oxygen use and metabolism (dysoxia). Intestinal oxygen uptake usually remains constant over a wide range of blood flows and pressures, with cellular function being substantively compromised when ischaemia leads to a >50% decline in intestinal oxygen consumption. Restoration of perfusion and oxygenation provokes additional injury, resulting in mucosal damage and disruption of intestinal barrier function. The primary cellular mechanism for sensing hypoxia and for activating a cascade of cellular responses to mitigate the injury is a family of heterodimer proteins called hypoxia‐inducible factors (HIFs). The HIF system is connected to numerous biochemical and immunologic pathways induced by IRI and the concentration of those proteins increases during hypoxia and dysoxia. Activation of the HIF system leads to augmented transcription of specific genes in various types of affected cells, but may also augment apoptotic and inflammatory processes, thus aggravating gut injury.

**Key points:**

- During intestinal ischaemia, mitochondrial oxygen uptake is reduced when cellular oxygen partial pressure decreases to below the threshold required to maintain normal oxidative metabolism.
- Upon reperfusion, intestinal hypoxia may persist because microcirculatory flow remains impaired and/or because available oxygen is consumed by enzymes, intestinal cells and neutrophils.

## Graphical abstract

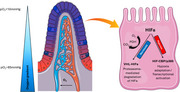

## 1 INTRODUCTION

The gastrointestinal tract is among the largest organs of the body and its primary function is absorption of nutrients. Uniquely among organs, the gastrointestinal tract is routinely exposed to copious pathogens and its ability to constrain pathogens depends critically on adequate tissue oxygen delivery and consumption. In recent years, there has been substantial progress in understanding the mechanisms by which reduced oxygen supply and use contributes to development of intestinal injury.

Normal hypoxia is defined by a physiologic state of well‐compensated low partial pressure of oxygen. In contrast, pathological hypoxia is defined by abnormally low oxygen partial pressure due to inadequate supply. Dysoxia, a related condition, is defined by abnormal tissue metabolism and oxygen use despite adequate oxygen supply. Pathological hypoxia and dysoxia result from systemic, cellular and subcellular cascades that trigger intricate inflammatory and oxidative responses; collectively, they are described as ischaemia–reperfusion injury (IRI).

IRI is a multifaceted process that includes an exaggerated oxidative stress response leading to tissue injury and systemic inflammatory response.^1^, ^2^ Here, we discuss the decompensated low oxygen use that accompanies intestinal IRI. We also review normal intestinal oxygenation and the aetiology and pathobiology of intestinal IRI, highlighting the effects of hypoxia and dysoxia and the pathways they promote. And finally, we present advances in detection and treatment of intestinal hypoxia and dysoxia.

## 2 OXYGEN STATE OF NORMAL INTESTINE

The gastrointestinal tract is heterogenous with respect to consumption of energy, distribution of perfusion and cellular oxygenation of its layers (mucosa, submucosa, muscularis and serosa). The intestinal epithelial cells have the highest energy requirement within the gastrointestinal tract, presumably consequent to their absorption function. The second highest energy requirement is observed in smooth muscle cells during generation of peristaltic waves.^3^, ^4^, ^5^, ^6^ A consequence of this high energy requirement is that the gastrointestinal tract receives 20–25% of the total cardiac output in the unfed state,^7^ and even more after feeding.^8^

The distribution of perfusion across intestinal layers unsurprisingly follows energy consumption, with 70–80% of total flow dedicated to the mucosa, about 5% to the submucosa and 15–25% to the muscular and serosal layers.^9^ Intestinal blood flow may be heterogenous even within the same region as oxygen supply is matched to local demand by modulating flow through pre‐capillary arterioles. Unlike most tissues, both arterioles and capillaries contribute to oxygen exchange and diffusion, resulting in more uniform delivery than would be possible with only a single source.^10^, ^11^

Distribution of intestinal oxygenation is also heterogenous on both macrostructural and microstructural levels. Macrostructural heterogeneity refers to the oxygenation gradient which progresses from the well‐oxygenated stomach to the small intestine and then to the large intestine which is least oxygenated.^12^, ^13^ On the microstructural level, there is an oxygenation gradient between basal and luminal epithelium within each tissue, with luminal tissues being least oxygenated. For example, the partial pressure of oxygen of the mucosa of small intestine villi is much lower than the pressure of the mucosa of the small intestine crypts, typically **<**10 mmHg versus about 85 mmHg.^14^, ^15^, ^16^, ^17^ The gradient apparently results from arterioles which enter the lamina propria in basal regions and release oxygen via capillary diffusion. Consequently, blood is less oxygenated by the time it reaches luminal structures.^18^, ^19^

The intestinal mucosa is intriguing from a tissue oxygenation perspective in that a substantial portion of the intestinal epithelial cells are normally relatively hypoxic.^20^ For example, oxygen partial pressure is usually 100–110 mmHg in lung alveoli, 20–60 mmHg in the renal cortex and 10–30 mmHg in the renal medulla.^21^ In contrast, the most luminal aspect of healthy colon tissue normally has an oxygen partial pressure < 10 mmHg.^22^, ^23^, ^24^ Nevertheless, many tissues function normally at oxygen concentrations equivalent to an atmosphere with just 5% oxygen, and some at partial pressures corresponding to just 1% oxygen.^21^

**FIGURE 1:**
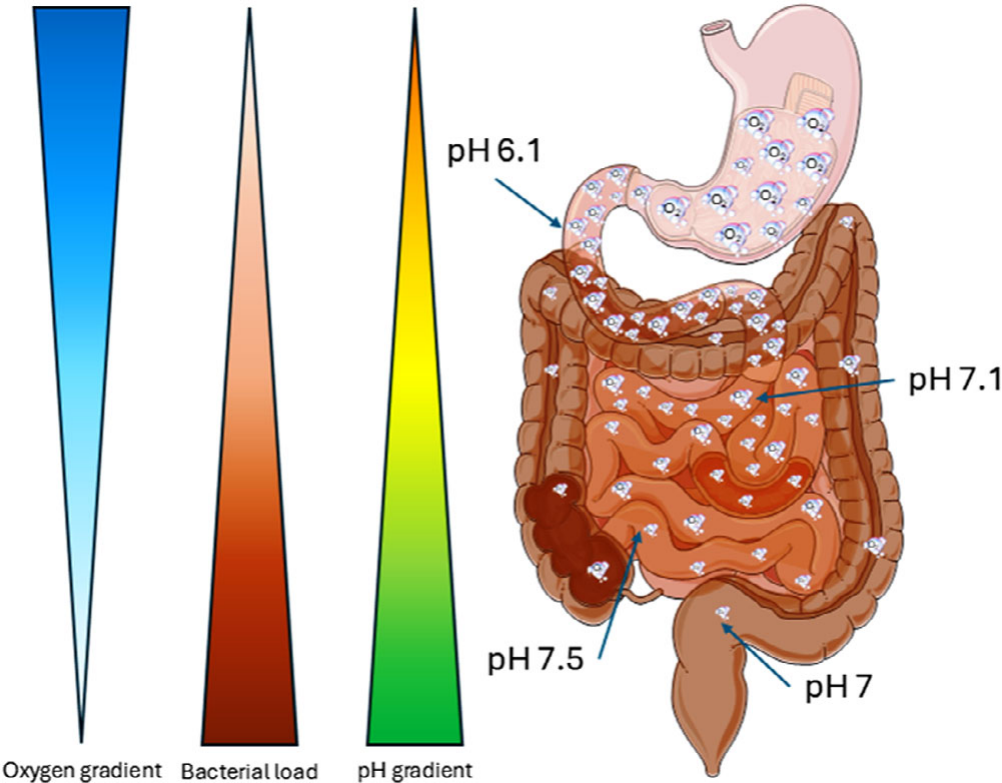
Physiological differences in oxygen tension, bacterial load and pH along the length of the small and large intestine. This development contributes both to the establishment of mucosal hypoxia and to the creation of a luminal anaerobic environment. The latter allows the rapid growth of obligate anaerobes, which form the mature microbiota of the distal gastrointestinal tract.

Intestinal physiologic hypoxia results from a combination of factors. As stated previously, oxygen from arterial blood diffuses to adjacent venules along the crypt villus axis resulting in progressively lower oxygenation along the radial axis from submucosa to the lumen.^25^ A formerly overlooked contributor is gastrointestinal tract microbiota which consume luminal oxygen and contributes to mucosal hypoxia. A consequence is a luminal anaerobic environment that allows growth of obligate anaerobes which are the most common bacteria in the distal gastrointestinal tract (Figure 1).^26^ The most frequently encountered species are Bacteroidetes, Clostridia, Actinobacteria and Fusobacteria.^27^, ^28^

## 3 AETIOLOGY OF INTESTINAL ISCHAEMIA–REPERFUSION INJURY

The gastrointestinal tract is affected both by local and systemic conditions. Ischaemia and subsequent re‐oxygenation provoke common cellular responses, microscopic findings and clinical consequences that are referred as IRI.^29^ The most intuitively understood mechanism resulting in ischaemia is macrovascular occlusion and subsequent compromise or infarction of a part of the intestine. The compromise can affect the arterial bed or the venous outflow.^30^

Arterial occlusion is usually consequent to systemic embolism (e.g., cardioembolic event by valvular disease, infective endocarditis or ventricular aneurysm) with arterial thrombosis being the second most common aetiology.^31^, ^32^ But any complete or even partial stenosis that compromises blood flow by 75% or more provokes tissue and cellular injury by oxygen and nutrient deprivation.^3^, ^33^ Despite this general rule, and precisely because the perfusion of the gastrointestinal tract is not uniform and does not rely on a robust blood supply or collateral circulation, some areas such as the splenic flexure are particularly prone to ischaemia.^34^

A second physiologic mechanism that promotes IRI is systemic hypoperfusion affecting multiple organs, the defining characteristic of shock.^35^, ^36^, ^37^, ^38^ Intestinal ischaemia during systemic hypoperfusion is worsened by marginal autoregulatory blood flow mechanisms which contrasts with organs such as the heart and brain.^39^ Systemic hypotension is by far the most common cause of IRI, accounting for 95% of colonic ischaemia and 20% of acute mesenteric ischaemia.^32^, ^40^ Once again, the threshold of decrease of blood flow by 75% to a particular bowel part is considered to be the critical cutoff that leads to IRI.

A third mechanism leading to ischaemia and IRI is microvascular compromise. This mechanism pertains to primary pathophysiology affecting the arterioles, capillaries and/or venules and is most heterogeneous in aetiology. For example, mechanical injury due to trauma, incarcerated hernia, intussusception or volvulus leads to the exposure of vascular endothelial cells to abnormal physical forces and mechanical stimuli. Τhe cellular responses are translated into biochemical signals that induce/aggravate local oxidative stress, inflammation and coagulopathy, subsequently leading to tissue ischaemia.^30^, ^37^, ^41^, ^42^, ^43^

Systemic conditions afflicting the bowel vascular bed can also provoke similar phenomena without an overt mechanical aggravating factor; instead representing topical manifestation of a systemic disease. Hypercoagulable states such as acute COVID‐19^44^ and immunologic/inflammatory diseases such as vasculitis^45^ are good examples. All of the aforementioned mechanisms can coexist, and such combinations are seen in IRI associated with cardiopulmonary bypass, necrotising enterocolitis and intestinal transplantation.^35^

Although animal models have been indispensable to the study of mechanisms of IRI,^46^, ^47^ research on intestinal hypoxia models remains limited (Table 1). The methodology used to provoke intestinal IRI includes procedural interventions mainly surgical clamping of the anterior mesenteric artery or aggressive haemodilution via the substitution of removed blood with isotonic fluid.^46^, ^47^, ^48^, ^49^ Other methods include administration of chemical compounds, such as oxidising agents, to induce the conversion of haemoglobin into methaemoglobin and environmental manipulation with aggressive decrease of the inhaled oxygen partial pressure.^46^ Shock provocation methods with cardiac arrest induction and haemorrhagic shock induction have also been reported.^50^, ^51^ Currently, there is no universally accepted standard for assessing the degree of hypoxia, with clinical assessments often distinguishing only between mild and moderate levels. Inadequate assessment methods pose challenges for modelling and evaluation which is worsened by the complexity and heterogeneity of the intestine.

**TABLE 1:** Current models of intestinal ischaemia/hypoxia and ischaemia–reperfusion injury.

Animal models of intestinal hypoxia			
Types	Construction principle	Main construction methods	Characteristics
Circulatory intestinal hypoxia,^47^ ^52^, ^53^, ^54^, ^55^, ^56^, ^57^, ^58^, ^59^, ^60^, ^61^, ^62^, ^63^, ^64^	Blocking the superior mesenteric artery to reduce blood flow to the intestines	Non‐traumatic vascular clamping or ligation of the artery	High reliability; requires advanced techniques and can induce systemic damage if prolonged
Chemical intestinal hypoxia^65^, ^66^	Conversion of haemoglobin into methaemoglobin to decrease oxygen supply	Oral administration of a strong oxidising agent, sodium nitrite	Convenient monitoring with minimal trauma; requires sophisticated equipment
Environmental intestinal hypoxia^67^, ^68^, ^69^, ^70^	Creating a low‐pressure, low‐oxygen environment (e.g., high‐altitude conditions)	Simulating high‐altitude using hypobaric chambers	Easy to operate and repeatable; it is expensive and can cause extra‐gastrointestinal issues

In vitro hypoxia models for intestinal cells			
Types	Construction principle	Main construction methods	Characteristics
Physical method^71^, ^72^, ^73^, ^74^	Creating a sealed hypoxic environment to isolate from external oxygen sources	1. Tri‐gas incubator method: Cells are placed in a tri‐gas incubator set to specific oxygen levels and hypoxia duration	Easy to operate, good reproducibility; cells may undergo reoxygenation after removal from incubator
		2. Anaerobic Bag Method: Uses an anaerobic bag system to create a hypoxic environment within a sealed container	Simple equipment, easy to operate; cannot precisely monitor oxygen levels
Chemical method^75^, ^76^, ^77^, ^78^, ^79^, ^80^, ^81^, ^82^, ^83^, ^84^, ^85^, ^86^	Inducing cellular hypoxia using chemical agents	1. Cobalt chloride method: Cobalt ions replace iron in prolyl hydroxylase, inhibiting HIF degradation and mimicking hypoxia	Easy to operate, low cost, good reproducibility; potential hazards to cells or humans
		2. Sodium hydrosulphite method: rapidly reduces dissolved oxygen in culture medium	Easy to operate, low cost; potential hazards to cells or humans
		3. HIF modulators: stabilise HIF by inhibiting prolyl hydroxylase, simulating hypoxia‐induced signalling pathways	Simple operation, good reproducibility; non‐oxygen‐dependent pathways may affect model outcomes

In vitro hypoxia models for intestinal organoids			
Species	Hypoxia induction methods	Organoid origins	Main evaluation indicators
Human^87^, ^88^, ^89^, ^90^, ^91^	Hypoxic environment (1% O_2_, 5% CO_2_, 94% N_2_) for 24h	Pluripotent stem cells	Permeability, antimicrobial peptides (AMPs), IL‐6, IL‐8, VEGF
	Hypoxia (less than 1% O_2_) for 12 h; reoxygenation for 30/120 min	Small intestine crypts	Morphology, lysozyme, IFABP, VEGFA, HIF‐1α
	1% O_2_ hypoxic environment for 24/48 h	Isolated small intestine crypts	Barrier function, Ki67, cleaved caspase 3, ZO‐1, HIF‐1α
	Hypoxic environment (2% or 20% O_2_, 5% CO_2_) for 9 days	Intestinal epithelial organoids from the colon	RNA sequencing; single‐cell RNA‐sequencing; neutrophile gelatinase‐associated lipocalin
Mouse^52^, ^55^, ^92^	Anaerobic environment for 12 h; reoxygenation for 4 h	Isolated ileum crypts	Morphology, surface area, budding number, HE staining, ZO‐1, occludin, Ki67
	5% O2 environment for 48 h	Isolated C57BL/6 small intestine	Morphology, surface area, IL‐6, TNF‐α

Experimental details of in vivo ischaemia–reperfusion models			
Species	Type of experimental IRI^a^	Segment of intestine	Characteristics
Cat^5^, ^93^, ^94^, ^95^, ^96^, ^97^, ^98^, ^99^, ^100^, ^101^, ^102^, ^103^, ^104^, ^105^, ^106^, ^107^, ^108^, ^109^, ^110^, ^111^, ^112^, ^113^, ^114^, ^115^	SMA^b^ complete occlusion	jejunum and ileum, ileum	In the low‐flow ischaemia model, blood flow is reduced to 20% of baseline levels (∼25–35 mmHg); The majority of injury associated with this model has been attributed to reperfusion as a result of increases in XOD^c^ and neutrophil activation, making the ischaemic event more of a priming mechanism for subsequent injury; This may or may not adequately represent clinical scenarios, in which injury caused by ischaemia may be so severe that reperfusion injury is of limited significance.
	Mesenteric vascular occlusion	Not specific	
	Low‐flow SMA^b^ occlusion	Jejunum, jejunum and ileum, ileum	
Dogs^116^, ^117^, ^118^, ^119^, ^120^	SMA^b^ occlusion Mesenteric vascular occlusion	Ileum Jejunum	Within a single animal, multiple loops can be subjected to varying lengths of IRI^a^; has the advantage of providing multiple treatment groups and controls within a single animal.
Mice^121^, ^122^, ^123^, ^124^, ^125^, ^126^, ^127^, ^128^, ^129^	SMA^c^ occlusion SMA^b^ occlusion^d^	Jejunum, jejunum and ileum, colon Jejunum, jejunum and ileum, other	Relatively low cost, ease of maintenance, rapid reproduction rate, mice are highly amenable to genetic manipulation
	SMA^b^ occlusion and collateral vessel ligation	Ileum	
Rats^4^, ^57^, ^123^, ^130^, ^131^, ^132^, ^133^, ^134^, ^135^, ^136^, ^137^, ^138^, ^139^, ^140^, ^141^, ^142^, ^143^, ^144^, ^145^, ^146^, ^147^, ^148^, ^149^, ^150^, ^151^, ^152^	Mesenteric vascular occlusion	Proximal colon	The degree of IRI^a^ following mesenteric vascular occlusion is variable. The colon appears to be more resistant to reperfusion injury compared with the small intestine Susceptibility to injury along the length of the small intestine is variable, with the ileum appearing to be more resistant to reperfusion injury than the jejunum
	SMA^b^ occlusion	Jejunum, jejunum and ileum, ileum, proximal colon	
	SMA^b^ and jejunal and colic artery occlusion	Jejunum, ileum	
Swine^153^, ^154^, ^155^, ^156^, ^157^, ^158^, ^159^, ^160^, ^161^, ^162^, ^163^, ^164^, ^165^, ^166^, ^167^, ^168^, ^169^, ^170^, ^171^, ^172^, ^173^, ^174^, ^175^, ^176^	SMV occlusion	Jejunum, ileum, colon	Share a similar pattern of XOD^c^ expression as humans Relatively small population of resident neutrophils Advantageous for models requiring surgical manipulation
	Mesenteric vascular occlusion	Jejunum and ileum, ileum	
	SMA^b^ embolism	Jejunum and ileum, small intestine and colon, other	
Human^130^, ^131^, ^177^, ^178^, ^179^, ^180^	Mesenteric vascular occlusion	Jejunum, proximal colon	Lack mucosal XOD^d^ at birth, and levels remain low even into adulthood; Have a relatively small population of resident neutrophils

^a^ Ischaemia–reperfusion injury.

^b^ Superior mesenteric artery.

^c^ Xanthine oxidase.

^d^ Transgenic animal use.

## 4 BIOLOGY, PATHOPHYSIOLOGY AND PATHOLOGY OF INTESTINAL ISCHAEMIA–REPERFUSION INJURY

### 4.1 Ischaemia

**FIGURE 2:**
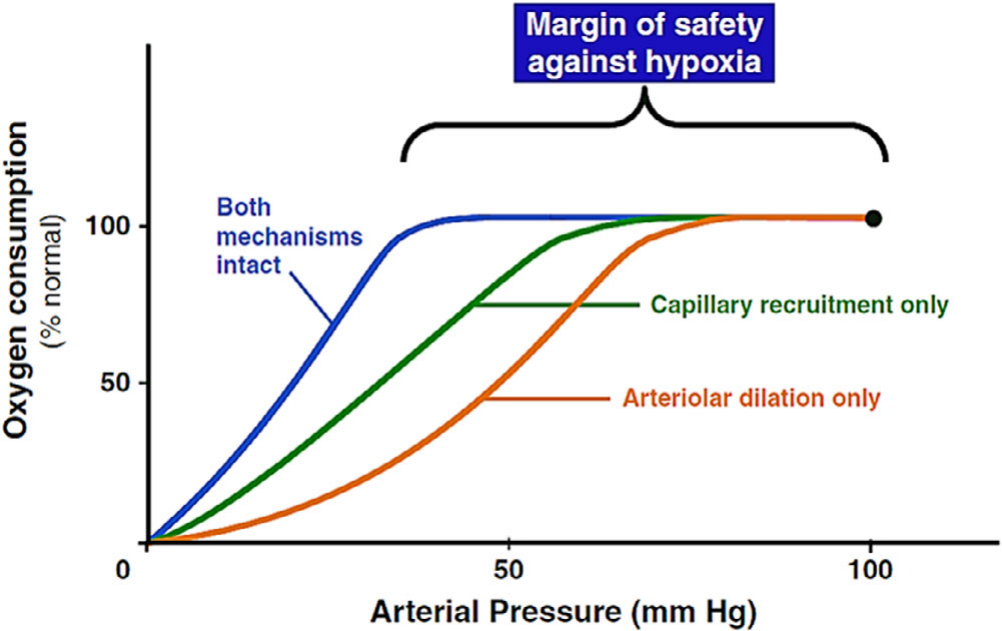
Roles of precapillary sphincter and arterial dilation in protecting the intestine against ischaemia when arterial pressure is reduced. When these mechanisms are intact, oxygen consumption is maintained until arterial pressure is reduced from 100 to 37 mmHg, providing a margin of safety against tissue hypoxia of 63 mmHg. If the arteriolar responses to hypotension are abolished, while precapillary sphincter responses (capillary recruitment) remain intact, oxygen consumption is maintained constant until arterial pressure is reduced to 65 mmHg. Hence, a 35‐mmHg margin of safety against tissue hypoxia is afforded by capillary recruitment alone. If, on the other hand, capillary recruitment is abolished and only arteriolar dilation is allowed to occur when arterial pressure is reduced, oxygen consumption is maintained constant until arterial pressure is reduced to 72 mmHg, indicating that arteriolar dilation provides a margin of safety against tissue hypoxia of 28 mmHg. Published with permission from Ref. 186. The rights in the material are owned by a third party.

Pre‐capillary sphincters and intestinal arterioles help sustain convective and diffusive delivery of oxygen to the tissue (Figure 2).^181^, ^182^, ^183^, ^184^, ^185^ The combination of these mechanisms provides substantial reserve when arterial pressure decreases, with recruitment of capillaries contributing more than arteriolar dilation.^186^ Collateral blood flow also helps prevent intestinal ischaemia and tissue hypoxia. There is nonetheless a tissue oxygenation threshold below which mucosal permeability is inversely related to oxygen tension, typically about 40–45 mmHg.^186^

IRI starts with ischaemia and evolves over the course of minutes to hours.^29^ At the molecular level, structural and functional changes in the mucosa have been observed after as little as 5–15 min of ischaemia. Ultrastructural studies of dog ileum suggest that intracellular mucosal damage (damage to the endoplasmic reticulum) occurs after 10 min of arterial occlusion.^186^ After 30 min of arterial occlusion, the intracellular spaces are widened, some epithelial cells have lifted from the basement membrane, and there is now overt cell failure characterised by mitochondrial vacuolisation, decreased oxygen uptake, loss of adenosine triphosphate and release of lysosomal enzymes.^186^ The most notable reversible microscopic finding of acute gastrointestinal ischaemia is called Gruenhagen's space and can be histologically identified in the small bowel villi and colonic surface epithelium within 30–60 min after injury onset.^3^, ^187^ It is created by the accumulation of leaked intracellular fluid from injured cells and products of the topical response by fibroblasts and myocytes of the adjacent lamina propria.^3^, ^177^, ^188^ Gruenhagen's spaces resolve when the ischaemic cascade stops sufficiently early.^177^ Arterial occlusions for two or more hours frequently produces massive epithelial lifting down the sides of the villi, completely denuded villi, disintegration of the lamina propria, increased mucosal permeability and net water loss into the bowel lumen.^186^

**FIGURE 3:**
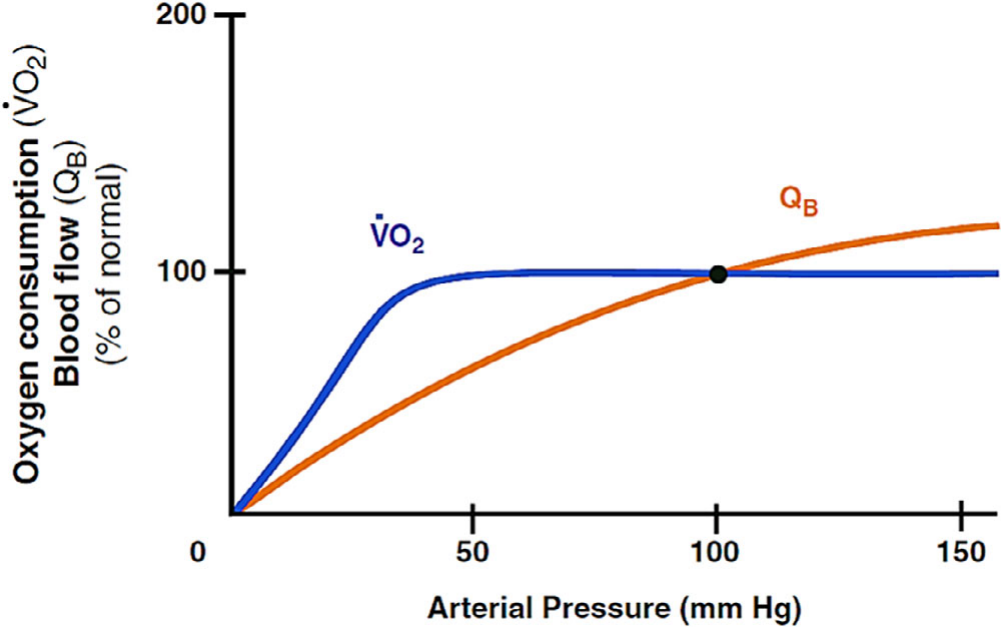
Dependence of intestinal oxygen consumption and blood flow on arterial pressure. Intestinal oxygen uptake usually remains constant over a wide range of blood flows and pressures, and it is compromised only when perfusion reaches below a critically low level. Published with permission from Ref. 186. The rights in the material are owned by a third party.

Although the ischaemic process is initially local and often reversible, a far more serious and often irreversible, change to the epithelium is disruption of its barrier function which is largely determined by oxygen availability and/or utilisation. Intestinal oxygen uptake usually remains constant over a wide range of blood flows and pressures and is only compromised when perfusion is below a critically level (Figure 3). In hypoxic conditions, microcirculatory oxygenation is heterogeneous, with well‐oxygenated microcirculatory units co‐existing units that are hypoxic because of microcirculatory shunting.

Pd‐porphyrin phosphorescence imaging and microsphere studies show, perhaps unsurprisingly, that hypoxic capillary units are found close to venules whereas oxygenated units are typically adjacent to arterioles.^189^ The relatively hypoxic units, naturally, are first to suffer and last to recover after IRI.^190^, ^191^ Shunting of oxygen from the microcirculation explains shock conditions such as haemorrhage and sepsis during which regional hypoxia/dysoxia is evident despite sufficient systemic oxygen delivery (Table 2).^192^, ^193^, ^194^

**TABLE 2:** Potential causes of intestinal dysoxia.

Cause	Associated mechanism
Hypoxemia	Hypoventilation, anaemia
Reduced blood oxygen delivery	Shock, regional reductions in blood flow, arteriovenous communications, increased affinity of haemoglobin for oxygen
Abnormal systemic capillary oxygen transport	Structural alterations or loss of systemic capillary bed
Abnormal interstitial oxygen transport	Altered oxygen diffusion through the interstitial space
Abnormal intracellular oxygen transport	Alterations of the intracellular compartment
Intrinsic mitochondrial disorders	Abnormal mitochondrial oxygen utilisation due to altered mitochondrial structure and/or function (mitochondrial disease)
Hyperoxia	Abnormally high oxygen tensions or oxygen content producing abnormal tissue oxygen utilisation
Thyroid disease	Increased oxygen consumption due to altered mitochondrial function (uncoupled mitochondria)
Hypervolemia, fluid overload, hypotonic solutions	Increased venous pressure, haemodilution, intracellular oedema, mitochondrial oedema
Intoxication	Dinitrophenol or salicylate intoxication (increased whole body oxygen consumption and mitochondrial uncoupling

Information is from Refs. 193 and 194.

Low perfusion states, microcirculatory shock and loss of autoregulation alters the relation between mitochondrial oxygen consumption and cellular oxygen partial pressure.^181^, ^182^, ^183^, ^195^, ^196^ Specifically, graded reductions in blood flow produce concomitant reductions in cellular oxygenation without altering mitochondrial oxygen uptake.^186^ The latter is reduced only when cellular oxygenation falls below a critical level; thereafter, low oxygen diffusion into cells decreases intracellular oxygenation to levels that no longer support normal oxidative metabolism.

**FIGURE 4:**
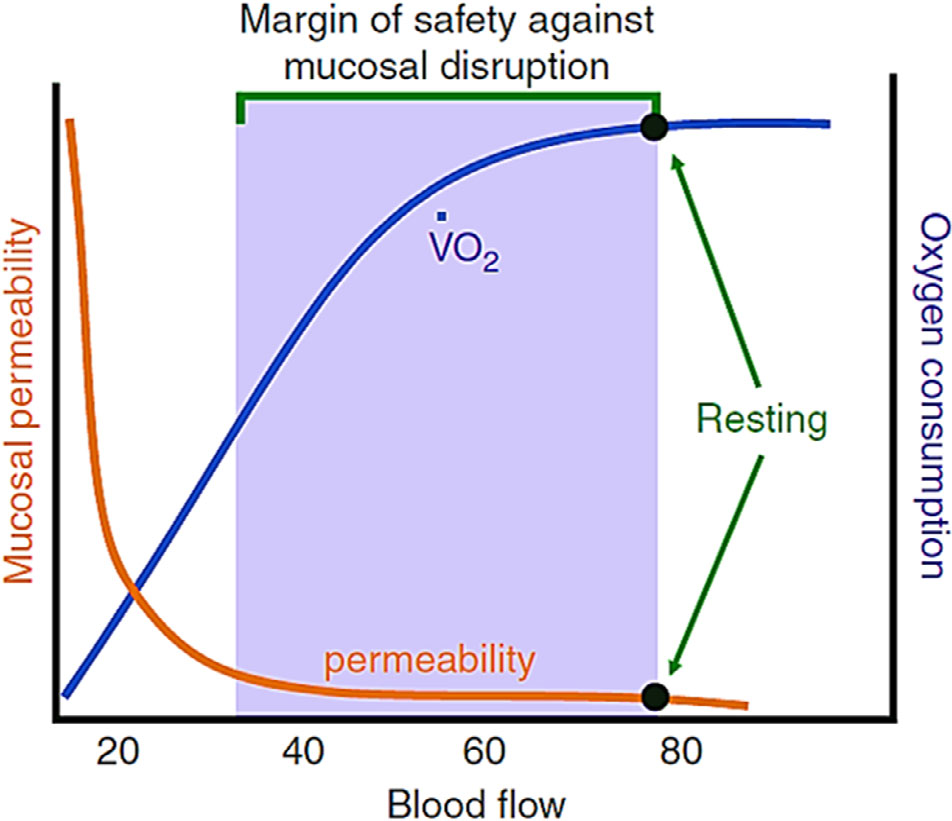
Relationship between intestinal mucosal permeability and oxygen consumption during reductions in blood flow. VO_2_, oxygen consumption. Published with permission from Ref. 186. The rights in the material are owned by a third party.

**FIGURE 5:**
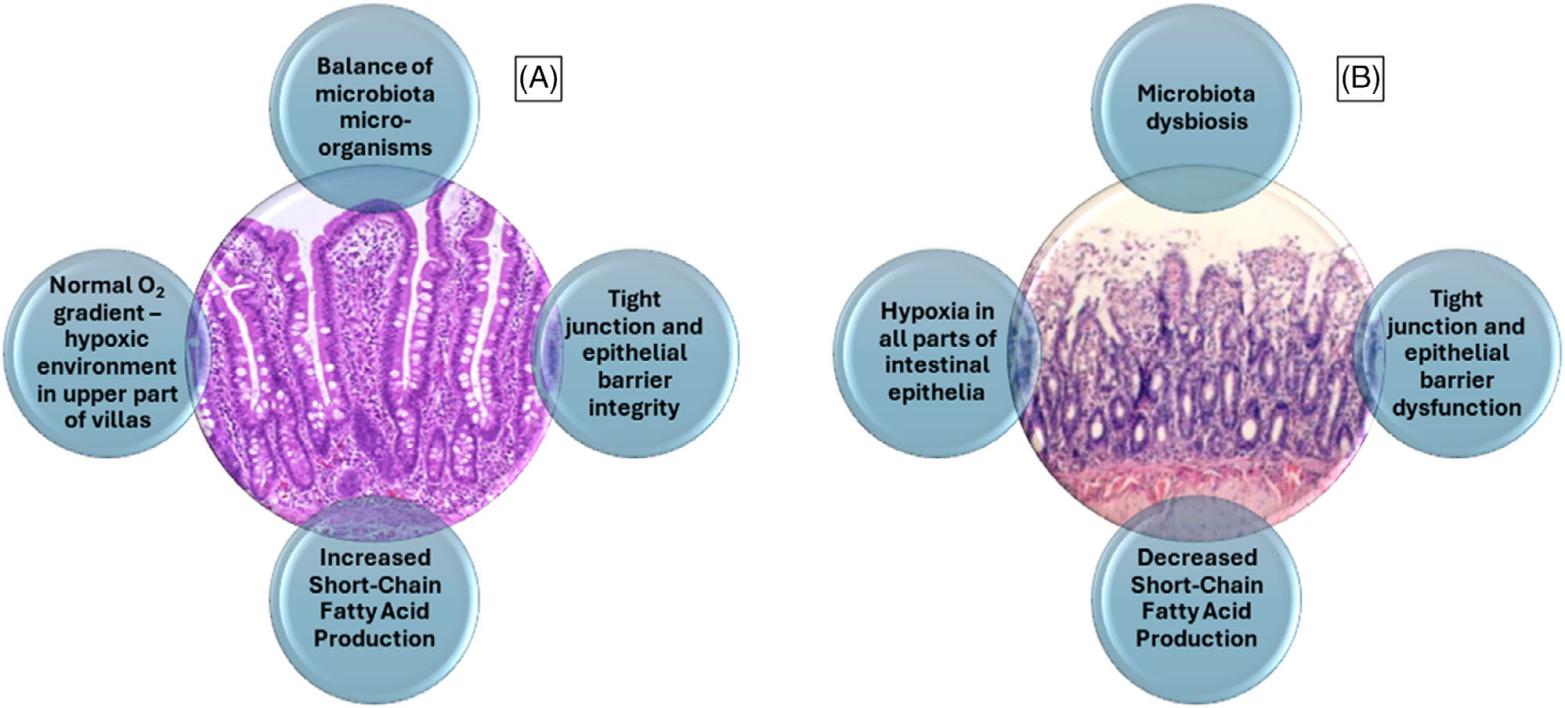
3D‐visualisation of the intestinal epithelial barrier in the physiological state (A) and after ischaemia and reperfusion (B). Low perfusion states, microcirculatory shock or loss of autoregulation that inhibits capillary recruitment alters the relation between mitochondrial oxygen consumption and cellular oxygen tension, leading to barrier disruption. Within the blue circles are presented the main characteristics of the healthy and reperfused barrier.

Barrier dysfunction depends on the integrity of intercellular junctions between the intestinal epithelial cells, mainly tight junctions, adherens junctions and desmosomes^197^—each of which is disrupted by sufficient ischaemia.^198^, ^199^, ^200^ Blood flow reduction leading to a >50% decline in intestinal oxygen consumption is accompanied by mucosal damage, depression of mucosal Na^+^/K^+^‐ATPase activity (which causes cell swelling and autolysis) and barrier dysfunction. The result is hypoxia/dysoxia‐induced mucosal permeability, with injury related to the reduction in oxygen consumption (Figures 4 and 5).^193^, ^201^, ^202^, ^203^

As ischaemia progresses, intestinal epithelial cells detach from each other and from the basal membrane, propagating from the luminal to the basilar/crypt cells as expected from the normal oxygen gradient. Cell death mediated by necrosis and apoptosis exacerbate injury, while insufficient clearance of dying cells leads to increased inflammation and impaired tissue repair.^47^ Loss of barrier function eventually results in shedding of the epithelial lining into the gut.^3^, ^4^, ^158^ A consequence is topical infiltration of luminally located toxins and bacteria which are part of the normal intestinal flora. Digestive enzymes produced by exocrine organs including the liver and pancreas also infiltrate and aggravate injury.^204^ The final manifestation of an ischaemic injury is dissemination of toxins and bacteria via the mesenteric lymphatics and consequent systemic inflammatory response, sometimes leading to multiple organ dysfunction.^30^, ^138^, ^205^, ^206^, ^207^

### 4.2 Reperfusion

**FIGURE 6:**
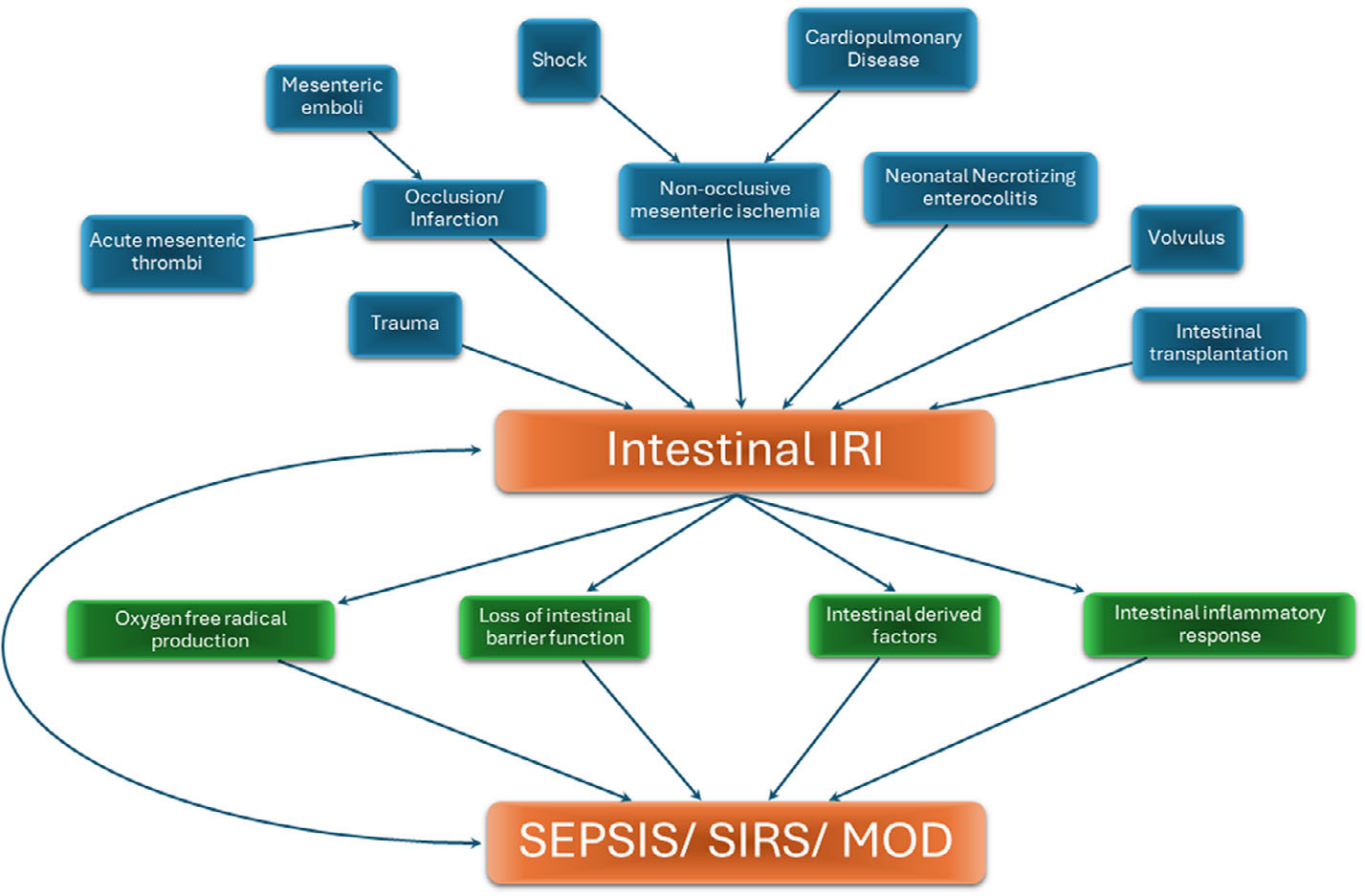
Aetiologic medical conditions, pathophysiological mechanisms and vicious circle components leading to intestinal ischaemia–reperfusion injury. IRI, ischaemia–reperfusion injury; SIRS, systemic inflammatory response syndrome; MODS, multiple organ dysfunction syndrome.

The progression of IRI does not conclude with correction of the initial causative pathophysiology; instead, restored perfusion and oxygenation provokes additional tissue injury.^29^, ^35^, ^190^, ^208^ When ischaemia is local, reperfusion is achieved by a spontaneous or therapeutic restoration of macrovascular and/or microvascular flow. In the case of systemic hypoperfusion, reperfusion occurs with the restoration of haemodynamic stability and/or resolution of conditions that compromise splanchnic perfusion.^32^, ^40^ The aetiologic factors along with processes implicated in IRI are depicted in Figure 6.

**FIGURE 7:**
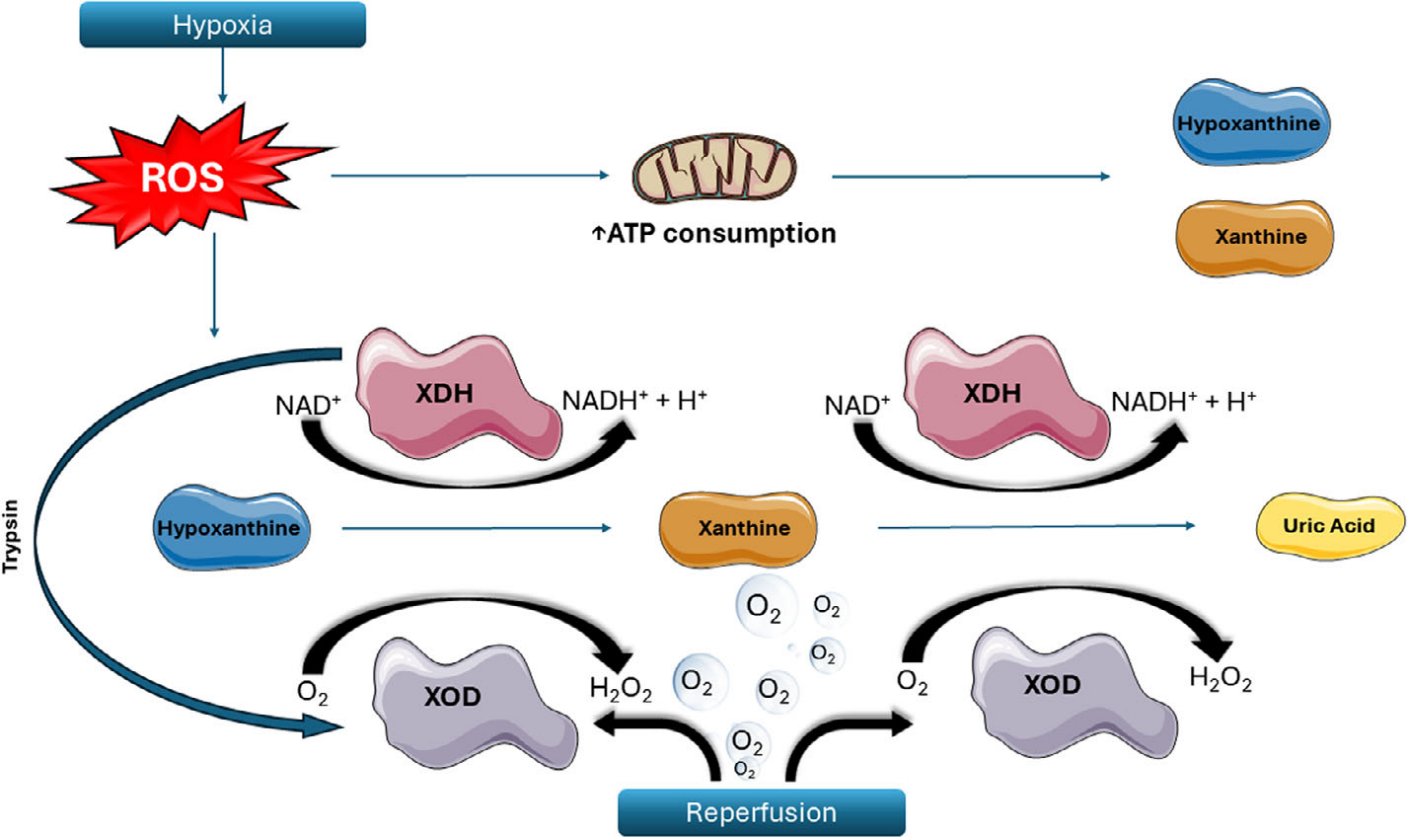
The biochemical basis for reperfusion injury includes the local production of reactive oxygen species and the conversion of xanthine dehydrogenase to xanthine oxidase. This figure illustrates the enzymatic conversion and oxidative stress response in ischaemic cells. Initially, in hypoxic environment the increased production of reactive oxygen species in ischaemic cells depletes adenosine triphosphate reserves, leading to the accumulation of hypoxanthine and xanthine. Under normal conditions, xanthine dehydrogenase catalyses the metabolism of these substrates using nicotinamide adenine dinucleotide to produce reduced nicotinamide adenine dinucleotide and uric acid. However, under hypoxic conditions, reactive oxygen species enhances the conversion of xanthine dehydrogenase into xanthine oxidase through irreversible proteolytic modification, predominantly directed by trypsin. Unlike xanthine dehydrogenase, xanthine oxidase uses molecular oxygen as a co‐factor, resulting in the production of hydrogen peroxide upon reoxygenation. The increase in intracellular hydrogen peroxide concentrations triggers further generation of oxygen‐based free radicals, exacerbating cellular oxidative stress. ROS, reactive oxygen species; ATP, adenosine triphosphate; NAD, nicotinamide adenine dinucleotide; XDH, xanthine dehydrogenase; XOD, xanthine oxidase; H_2_O_2_, hydrogen peroxide.

The biochemical basis for reperfusion injury is local production of reactive oxygen species.^5^, ^97^, ^98^, ^102^, ^104^, ^105^, ^106^, ^107^, ^108^, ^109^, ^110^, ^111^, ^125^, ^209^, ^210^, ^211^, ^212^, ^213^, ^214^, ^215^ Reactive oxygen species are generated when ischaemic cells consume available adenosine triphosphate reserves and then produce metabolism byproducts including hypoxanthine and xanthine.^216^ The enzyme xanthine dehydrogenase is a normal part of purine metabolism and uses nicotinamide adenine dinucleotide as a co‐factor to produce reduced nicotinamide adenine dinucleotide and uric acid. But in hypoxic conditions, xanthine dehydrogenase undergoes irreversible proteolytic modification, mostly by trypsin, and converts to xanthine oxidase which uses the same substrates as xanthine dehydrogenase but consumes oxygen as a co‐factor to produce hydrogen peroxide.^107^, ^217^ Upon restoration of oxygenation, xanthine oxidase metabolises the newly abundant oxygen which markedly increases the intracellular concentration of hydrogen peroxide. Accumulation of peroxide leads to the enzymatic and non‐enzymatic generation of several other oxygen‐based free radicals (Figure 7).^96^, ^97^, ^211^, ^212^

Activation of nicotinamide adenine dinucleotide phosphate oxidases, a family of integral enzymes found in many tissues including intestine, results in an active protein complex that also produces extracellular hydrogen peroxide.^218^, ^219^, ^220^, ^221^ Furthermore, nitric oxide synthase functions as a homodimer and produces nitric oxide under normal conditions. But when the available tetrahydrobiopterin is exhausted, such as during oxidative stress induced by xanthine oxidase and nicotinamide adenine dinucleotide phosphate oxidases, the molecules uncouple and produce hydrogen peroxide instead of nitric oxide.^202^, ^222^ Hydrogen peroxide accumulation from various sources and subsequent reactive oxygen species production is deleterious both by disrupting intracellular structures and via strong chemoattraction of neutrophils.

An additional source of reactive oxygen species during reperfusion is mitochondrial dysfunction. The initial steps include disruption of normal electron transport chain function, most importantly complex I, exhaustion of antioxidant mitochondrial capacity, generation of superoxide anion and its rapid transformation to hydrogen peroxide, opening of mitochondrial permeability transition pore (mPTP) and intracellular release of hydrogen peroxide and cytochrome c.^218^, ^223^ In an environment rich in reactive oxygen species, mitochondrial DNA which is normally located in the mitochondrial matrix is released into the cytoplasm via the mPTP or via vesicle formation and cytoplasmic release.^224^, ^225^, ^226^, ^227^, ^228^ Mitochondrial DNA functions as a damage‐associated molecular pattern and its presence in the cytosol is implicated in the activation of several cellular pathways, analysed below.

Aggressive infiltration of affected tissues by neutrophils and their inflammatory activity is the most prominent immunologic component of the reperfusion phase of intestinal IRI. The first step in this process is chemotactic and cytokine‐mediated attraction of the inflammatory cells. Attraction is mediated by various compounds. First, cytoplasmic mitochondrial DNA activates the endoplasmic Toll‐like receptor‐9,^229^ which usually functions as a pathogen recognising system in intestinal epithelial cells.^230^, ^231^ Activated receptors binds myeloid differentiation factor 88 and the complex further activates the NF‐kB pathway which provokes release of cytokines.^229^ Second, increased cytosolic concentration of reactive oxygen species and the cytosolic presence of mitochondrial DNA activate the nucleotide‐binding domain and leucine‐rich repeat pyrin 3 domain inflammasome which has been identified in both intestinal epithelial cells and intestinal immune cells.^232^, ^233^ Inflammasome formation causes caspase 1 activation in multiple cellular pathways including ones that release IL‐1β and IL‐18.^234^ These cytokines are released along with other systemically proinflammatory mediators including transcription factors, hypoxia‐inducible factor (HIF)‐1, cyclooxygenase‐2 and poly(ADP‐ribose) polymerase to attract neutrophils to the affected intestine.^235^, ^236^, ^237^, ^238^ Digestive enzymes, toxins and bacteria that cross the compromised mucosal barrier also contribute.^179^, ^239^, ^240^

**FIGURE 8:**
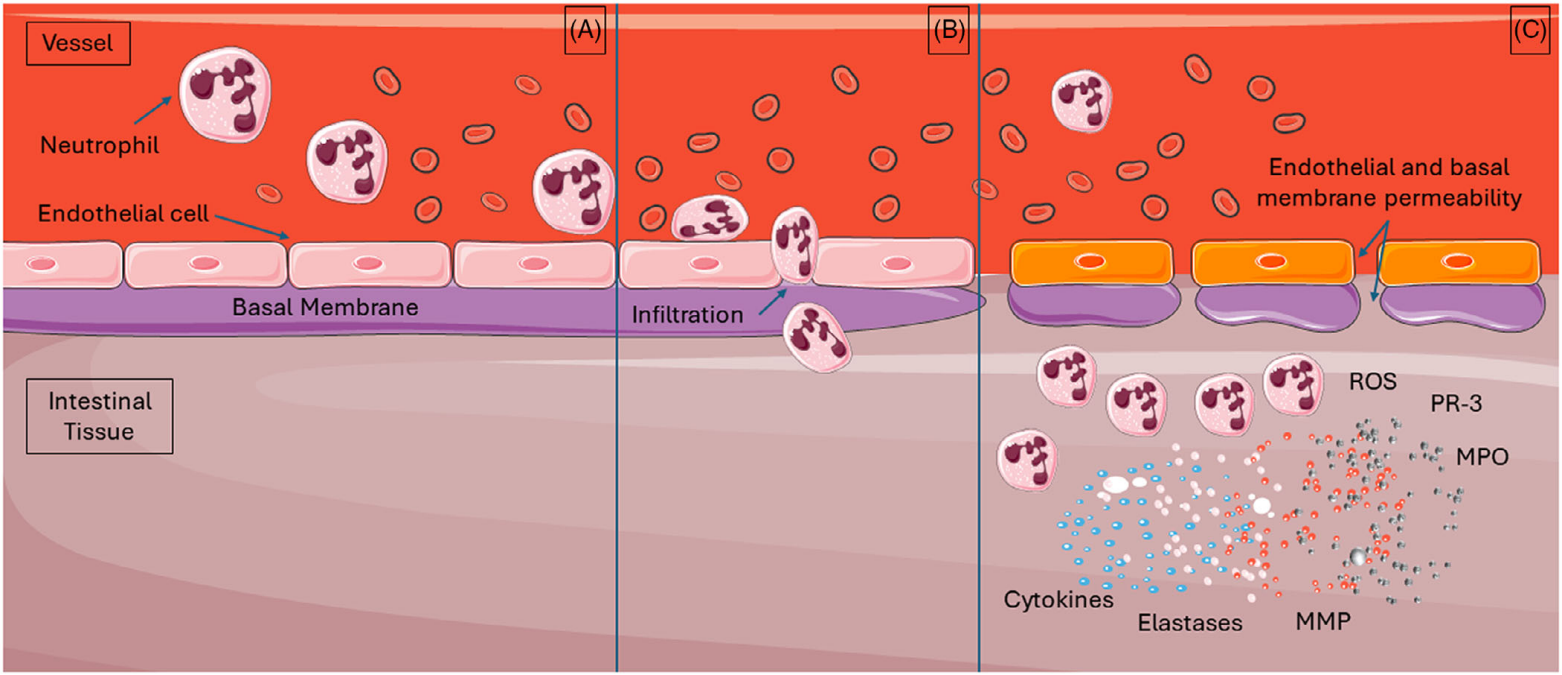
Infiltration of neutrophils during the reperfusion phase of intestinal IRI. Initially, chemokines and cytokines recruit neutrophil to intestinal tissues (A). Upon attraction, neutrophils infiltrate the affected tissue through gaps they create between the endothelial cells and through the endothelial basal membrane, significantly increasing the permeability of the capillaries (B). Activated neutrophils release chemical compounds, notably reactive oxygen species and enzymes such as elastase, myeloperoxidase, protease‐3 and metalloproteinases. ROS, reactive oxygen species. MPO, myeloperoxidase; PR‐3, protease‐3.

Neutrophils infiltrate affected tissue through gaps they create between the endothelial cells and through the endothelial basal membrane, substantially increasing capillary permeability.^163^ Neutrophil accumulation worsens tissue hypoxia because these metabolically activated cells demand much oxygen.^241^ But the most deleterious effect of neutrophils is their inherent cytotoxicity which includes the release of chemical compounds, notably reactive oxygen species^107^, ^112^, ^125^ and enzymes such as elastase, myeloperoxidase, protease‐3 and metalloproteinases (Figure 8).^110^, ^114^, ^242^ The putative purpose of releasing these compounds is to kill bacteria and other invading pathogens, but they also cause collateral damage to the extracellular matrix and surrounding cells.

Another contributor to IRI is microcirculation. Disruption of capillary and venule walls by neutrophilic infiltration causes perivascular oedema and endothelial injury which creates external pressure on micro‐vessels. Swollen endothelial cells reduce luminal diameter and microvascular blood volume, a phenomenon that is well described in the cardiac circulation.^108^, ^243^, ^244^ Consequent reduction in local blood flow decreases the relative haematocrit in small vessels (Fåhræus effect; Table 3)^245^, ^246^ and increases endothelial shear stress.^43^ Intravascular thrombus formation and exacerbated local hypoperfusion is aggravated by neutrophil clustering within capillaries, platelet aggregation, exposure to various tissue‐based coagulation factors and subsequent activation of the coagulation cascade.^99^, ^247^

**TABLE 3:** Reduction of the haematocrit by reducing the microvessel diameter.

	Blood volume		
Microvessel diameter (mm)	Microvessel Hct (%)	Plasma (%) (1 – microvessel Hct^a^)	Average velocity of RBCs^b^, ^c^
1.100	40.5	59.5	100
0.750	40.1	59.9	101
0.450	39.8	60.2	103
0.250	39.2	60.8	106
0.095	33.6	66.4	135
0.050	28.0	72.0	175

The table gives the change in the volumetric relationship between the erythrocytes and the plasma, when blood from a healthy person is streaming through microvessels of different diameter, and the calculated average velocities of the erythrocytes in proportion to those of the plasma. With decreasing diameter of the microvessels below 0.1 mm the relative erythrocyte volume is very rapidly decreasing, while the velocity of the erythrocytes in proportion to that of the plasma very rapidly increasing.

Modified from Ref. 245.

^a^ Haematocrit.

^b^ Red blood cell.

^c^ That of plasma = 100.

Red blood cells also contribute by releasing ATP, nitric oxide and S‐nitrosothiols. During deoxygenation, haemoglobin reacts with nitrite to form nitric oxide, but it also has a high affinity for nitric oxide. Scavenging of nitric oxide by haemoglobin can cause vasoconstriction, which is greatly enhanced by extracellular haemoglobin.^248^ And finally, low red cell volumes promote deformation and folding,^249^, ^250^ whereas high red cell volumes promote diffuse trajectories and shear.^251^ Both derangements decrease delivery of oxygen to intestinal cells.

Many mechanisms thus conspire to seriously compromise local perfusion during ischaemia–reperfusion and create vicious cycles that can prevent recovery.

## 5 HYPOXIA AND DYSOXIA IN INTESTINAL IRI

### 5.1 Regulation and function of HIFs

The linchpin of cellular responses to hypoxia is a group of transcription factors called HIFs. Initial studies of HIF evaluated the renal response to hypoxia and consequent production of erythropoietin which is mediated by HIF system activation.^252^, ^253^, ^254^, ^255^ HIFs have a heterodimer structure with an alpha and a beta subunit.^256^ The intracellular concentration of the active complex depends on the rate of hydroxylation of the alpha subunit and subsequent funnelling of the entire molecule through a ubiquitination and degradation process mediated by the von Hippel–Lindau tumour suppressor protein.^257^, ^258^

**FIGURE 9:**
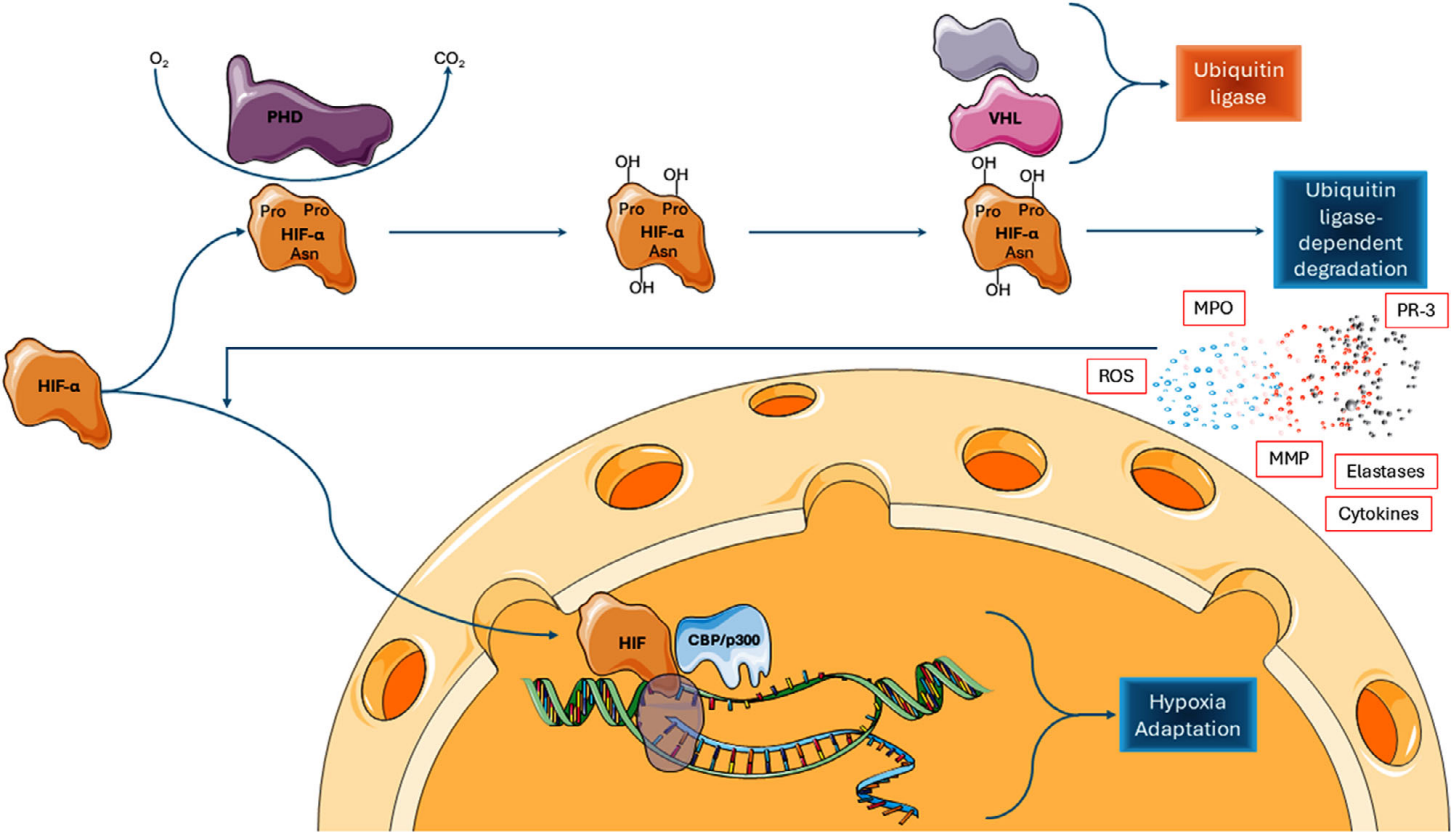
Oxygen‐dependent effects on the modulation and activation of hypoxia‐inducible factor‐α. Under normoxic conditions, HIF‐α undergoes degradation by prolyl hydroxylase domain‐containing proteins. In the hypoxic intestine, these processes are significantly suppressed and HIF‐a migrates to the nucleus where it binds to specific histone acetyl‐transferases (CBP and p300), altering the transcription of multiple genes. PHD, prolyl hydroxylase domain‐containing proteins; HIF‐α, hypoxia‐inducible factor‐α; VHL, von Hippel–Lindau tumour suppressor protein.

In normoxic conditions, HIF‐1α is degraded by hydroxylation at an oxygen‐dependent rate that is mediated by the enzyme family of prolyl hydroxylase proteins which use oxygen as a substrate. Thereafter, von Hippel–Lindau E3 ubiquitin ligase complex completes the breakdown of HIF to proteasomes.^259^, ^260^, ^261^, ^262^ Prolyl hydroxylase protein activity, and thus the hydroxylation and degradation of HIFs, is suppressed in hypoxic environments which leads to intracellular accumulation of HIFs. When concentrations increase sufficiently, HIFs migrate into the nucleus^263^ where they bind specific histone acetyl‐transferases (CBP and p300) and form a complex that enhances transcription of numerous genes.^264^, ^265^, ^266^, ^267^ HIF proteins are present in various types of cells, with HIF‐1 and HIF‐2 identified in intestinal epithelial cells, and in other intestinal cells including lymphocytes (Figure 9).^268^, ^269^

HIF concentration also depends on enzymes called factors inhibiting HIF which are the only known hypoxia‐sensitive asparagine hydroxylases. Post‐translationally, factors inhibiting HIF modify an asparagine residue in HIF‐1α, a change that prevents HIF‐1α from binding p300/CRB, thereby depressing HIF activity.^270^ Like prolyl hydroxylase domain‐containing proteins, factors inhibiting HIFs are less active under hypoxic conditions, thus enabling HIF‐1α to effectively engage with its co‐activators and enhance gene expression.

The sensitivity of prolyl hydroxylases and factors inhibiting HIF is expressed by the value of the Michaelis–Menten constant (*K*_M_), or the oxygen tension at which the reaction rate is half‐maximal.^271^ Given an average intracellular oxygen tension of 7–20‐mmHg (corresponding to 1.0–2.5% oxygen), factor inhibiting HIF (*K*_M_ = 50–80 mmHg/6.5–10.5% oxygen) may be more effective than prolyl hydroxylase (*K*_M_ = 120–210 mmHg/15.7–27.6% oxygen) as a physiological oxygen sensor.^271^

The HIF system is an integral mediator of the cellular response to hypoxia. For example, mitochondrially generated reactive oxygen species appear to increase intracellular concentration of HIFs, although the specific chemical pathway remains unclear.^272^ Carbon monoxide and ammonia similarly trigger the HIF cascade, eliciting the same physiological response.^273^ In contrast, nitric oxide, hydrogen sulphide and carbon dioxide inhibit the system.^273^

Immune mediators including tumour necrosis factor‐a and interleukin‐1b,^274^, ^275^, ^276^ bacteria and bacterial components such as lipopolysaccharides all reduce HIF deactivation, thereby increasing intracellular HIF concentrations—even in normoxic conditions.^277^, ^278^, ^279^ Many other proteins such as STAT3 and immunologic systems such as PI3K/AKT/mTOR, RAS/RAF/MEK/ERK and IKK/NF‐κB also modify the complex HIF cascade.^280^, ^281^, ^282^, ^283^ Consequently, the effects of HIF activation are multifaceted, only partially understood, and depend on the duration and activating mechanism.^284^

### 5.2 The effect of HIFs on intestinal ischaemia–reperfusion injury

Activation of the HIF cascade leads to structural and functional changes in intestinal epithelial cells. Structural changes include maintenance and augmentation of tight junctions. For example, HIF promotes expression of the CLDN1gene and then production of claudin‐1 protein which is a critical component of tight junctions. Functional changes include increased production and luminal secretion of mucus structural components (primarily mucins via augmentation of the MUC family genes) that stabilise mucus proteins such as intestinal trefoil family factor peptides and antimicrobial and immune mediating peptides including defensins, lysozyme and secretory phospholipase 2.^285^, ^286^, ^287^, ^288^ Combined, the net effect is to maintain tight junction integrity and mucosal barrier function, along with mucosal permeability under hypoxic conditions.^289^ However, HIF‐1 can also augment apoptotic and inflammatory processes which can aggravate IRI‐induced gut mucosal injury leading to barrier permeability, bacterial translocation and systemic inflammation.^284^ Which effect dominates depends on the circumstances and remains poorly understood.

The effect of hypoxia and increased HIF concentration on intestinal immune cells is also important. The main intestinal immune cells are T lymphocytes. ‘Conventional’ T cells (CD4‐ and CD8αβ^+^ cells) are located intra‐epithelially and in the lamina propria and mainly defend against infections. In contrast, ‘unconventional’ T cells (CD8αα^+^, CD8αα^−^, CD4^−^ and CD8αβ^−^), which are also localised intra‐epithelially, regulate immune responses and maintaining intestinal homeostasis.^290^ Activation of the HIF cascade has several effects on T cell function. First, it induces a shift from primarily using fatty acids when quiescent to intense glycolysis when activated. The shift is mediated by increased expression of the SLC2A1 gene, augmented function of the GLUT1 transporter which increases the intracellular availability of glucose, and activation of the genes encoding relevant glycolysis enzymes (including lactate dehydrogenase, pyruvate kinases and phosphofructokinase 1).^291^ The second less understood effect pertains to cytokine production, transition to effector cells and immune cell recruitment from secondary lymphoid tissues.^292^, ^293^ Available data suggest that an increase in HIF promotes activation, cytotoxicity and altered transition to memory cells^292^, ^294^—all of which can promote hypoxia‐induced intestinal injury.^295^, ^296^

Several other types of immune cells are affected by the HIF cascade activation including dendritic cells, macrophages and neutrophils—all of which are present in the gastrointestinal track. Again, the best investigated change observed in these cells is the switch from aerobic to glycolysis‐based metabolism via the same gene and protein upregulation described for lymphocytes. This shift constitutes an integral and critical step of immune cell activation, function and proliferation.^283^ Activation of HIFs in intestinal dendritic cells increases immune cell migration to affected tissues and maturation and antigen presentation. The mechanism appears to be upregulation of several cell membrane receptors, most prominently the Toll‐like receptor‐2 and ‐6 and the CCR7 surface receptor.^297^, ^298^, ^299^, ^300^ Changes to the dendritic cell immune mediator production under the influence of HIF activation remain under investigation and are not yet well understood.^283^

**FIGURE 10:**
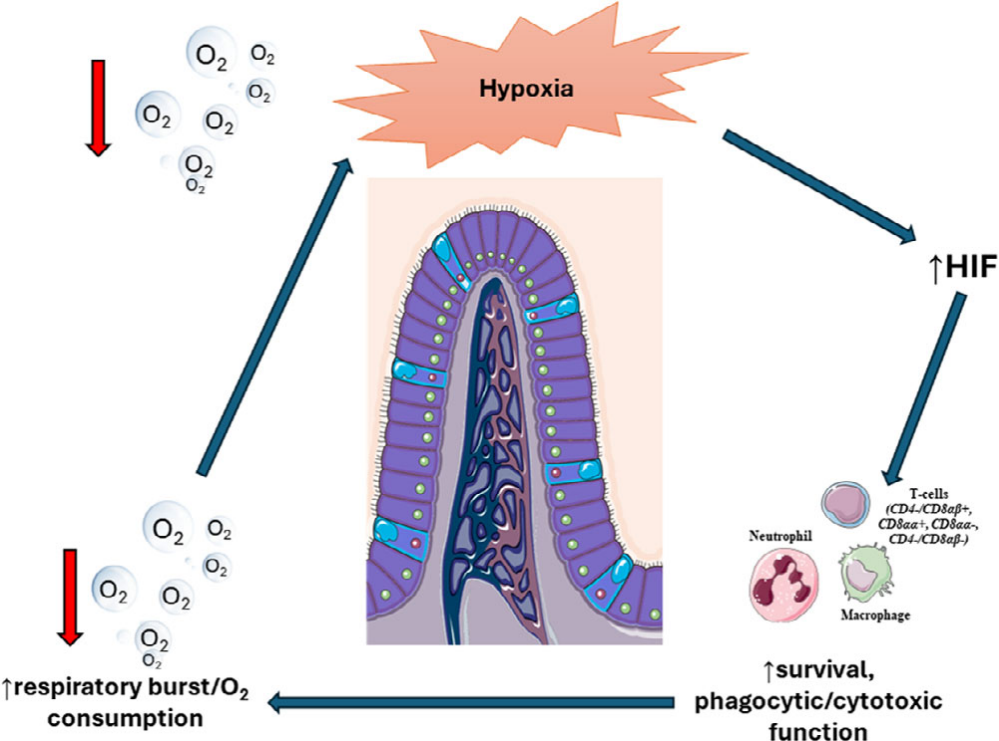
Immune cell adaptation in intestinal ischaemia–reperfusion injury. Under hypoxic or dysoxic conditions, activation of the HIF pathway leads to prolonged immune cell survival and increased phagocytic and cytotoxic function, as mediated by an augmentation of their respiratory burst and oxygen consumption. The latter exacerbates tissue hypoxia, which in turn further activates the HIF pathway in affected and surrounding intestinal tissues. HIF, hypoxia‐inducible factors.

HIF system activation polarises macrocytes toward their proinflammatory sub‐type which increases motility and bacteriophage activity.^301^ Apart from the metabolic profile switch to glycolysis, this process includes alteration of membrane receptor constitution, notably with the increase of CD40 and CD206 receptors and the production and excretion of several different cytokines including IL‐1β, TNF‐α and IL‐6.^302^, ^303^, ^304^, ^305^ Under IRI and hypoxic conditions, the HIF pathway prolongs neutrophil survival and increases phagocytic and cytotoxic function. A pronounced effect of HIF activation is to increase neutrophil survival by intensifying glycolysis which suppresses hypoxia‐induced apoptosis.^306^ Oxidative killing of bacteria requires a respiratory burst and consequent increase in oxygen consumption^307^ which exacerbates tissue hypoxia (Figure 10). Local hypoxia stabilises HIF transcriptional machinery^241^ which probably protects mucosa and helps resolve inflammation. Finally, HIF activation induces leukocyte β2 integrin expression, a critical structural and functional protein component for CD18 that mediates leukocyte adhesion and extravasation^308^ along with protein production.

Matrix metalloproteinases (MMPs) are zinc‐dependent proteinases which regulate many cellular activities and can degrade nearly all extracellular matrix components. MMPs are produced in abundance once reperfusion is established and play a central role in disease progression.^309^, ^310^ For example, HIF directly increases various metalloproteinases including MMP‐1,^311^ MMP‐15^312^ and MMP‐17.^313^ Reactive oxygen species also activate MMPs both directly as observed in cell‐free environments and indirectly as observed in experimental neutrophil‐rich environments.^314^, ^315^, ^316^

Disruption of mucosal barrier during IRI allows penetration of pancreatic trypsin into the intestinal wall.^317^ Trypsin is a strong activator of the MMP‐9 pro‐form that is released from neutrophils,^317^, ^318^ while MMP‐9 and myeloperoxidase play important roles in the physiological turnover of the extracellular matrix through degradation and remodelling.^319^ Overexpression of MMP‐9 during acute IRI stimulates inflammation and tissue injury and increases intestinal villous loss, particularly when MMP‐9 is upregulated by thiobarbituric acid reactive substances.^318^ MMP‐9 activity also enhances lipid peroxidation in the gastrointestinal tract and in other end‐organs including the kidney.^318^, ^320^

## 6 DETECTION OF INTESTINAL HYPOXIA AND DYSOXIA

Despite the importance of intestinal hypoxia and dysoxia, there are no routine diagnostic tools for evaluating the conditions. Assessment is mostly limited to ex vivo methods since organ systems respond heterogeneously to changes in systemic oxygen delivery. One approach for dysoxia is to evaluate the redox state of intestinal cells. For example, the a‐hydroxybutyrate to acetoacetate ratio is a marker of oxygen‐limited adenosine triphosphate flux in the liver,^321^ whereas the lactate‐to‐pyruvate ratio might be a more specific marker of dysoxia than lactate alone.^193^

Assessment is complicated because factors other than oxygen partial pressure matter. For example, experimental research suggests that hyperlactatemia, despite unchanged or increased tissue oxygen partial pressure, coincides with both reduced function and structural damage to intestinal mitochondria.^322^ But as clinicians and researchers well appreciate, there are many potential causes of elevated lactate so the clinical and prognostic importance of hyperlactatemia varies widely by disease state.^323^ Currently, there are no well validated biochemical markers of intestinal ischaemia.

Direct assessment approaches include intestinal tonometric measurement of mucosal pH and carbon dioxide (both gastric and colonic) and algorithmic estimates of tissue oxygenation. However, intestinal tonometry is tricky, invasive and evaluates a single local region that may may not be representative and can be perturbed by the measurements *per se*.^49^, ^324^, ^325^ Tissue reflectance spectrophotometry is another method of direct hypoxia assessment. The method has been used in various preclinical scenarios, including haemorrhage and sepsis over several decades, but does not obviously improve assessment and management of IRI.^326^

Oxygen sensors have been inserted into the tissue plane between the serosa and mucosa of descending colon to measure intramural intestinal oxygen partial pressure during surgery. Although the method may be helpful for direct hypoxia assessment, tissue injury at the intestinal sensor site is inevitable and may influence measurements. Furthermore, it is not currently possible to measure intestinal oxygenation after the abdomen is closed.^327^ Surface oxygen electrodes have been also used in intestinal oxygenation research. In theory, intestinal oxygen electrodes can estimate interstitial oxygen partial pressure,^328^ yet their penetrations for oxygen detection does not exceed 15 µm.^329^ Furthermore, insertion of oxygen electrodes causes tissue microtrauma, resulting in relatively low oxygen values.^330^

Oxygen‐dependent phosphorescence quenching has been used for in vivo measurement of microcirculatory oxygen tension.^331^ The method is based on the principle that a Pd‐porphyrin molecule excited by light can release the absorbed energy either as light (phosphorescence) or transfer the energy to oxygen molecules.^332^ This process causes the phosphorescence intensity and decay time to decrease in an oxygen‐dependent manner.^333^ Oxygen‐dependent quenching of phosphorescence is especially helpful during hypoxia since sensitivity increases as oxygen partial pressure decreases. Therefore, high oxygen partial pressures in arterioles generate short decay times and low phosphorescence intensities. The measurement penetration depth extends to 300–500 µm which far exceeds the <15 µm for oxygen electrodes and thus inherently provides better information about tissue oxygenation.^330^ However, this technique requires injection of a Pd‐porphyrin dye that limits its use to experimental animals. Although a phosphorescence‐based fibre optic oxygen sensor has been validated for clinical use, validated intestinal phosphorimeters are not yet commercially available.^334^

Reduced nicotinamide adenine dinucleotide fluorescence can assess the heterogenous nature of microcirculatory oxygenation and lack of oxygen in mitochondria.^335^ The technique is based on oxygen‐dependent quenching of protoporphyrin IX fluorescence. Fluorescence intensity thus becomes an indicator of a more reduced state of nicotinamide adenine dinucleotide and a decrease of the mitochondrial electron transfer chain activity. To measure nicotinamide adenine dinucleotide fluorescence, tissue is illuminated with ultraviolet light (310–380 nm) and the emitted fluorescence light is measured at 410–490 nm peaking at 450–460 nm.^336^ A limitation of this technique is that it is qualitative and relies on the detection of emitted fluorescence induced by light excitation at 360 nm which is highly absorbed by haemoglobin, thus limiting in vivo utility.^337^ On the other hand, the delayed fluorescence lifetime of endogenous protoporphyrin IX can measure oxygen tension at the mitochondrial level and enables mitochondrial respirometry in vivo.^338^ However, the concentration of endogenous protoporphyrin IX may be too low to perform reliable measurements and further research is needed to validate the method for intestinal measurements.

An interesting approach of indirectly identifying IRI sequelae is to evaluate changes in the microbiome. As described above, the normal colonic flora is comprised of obligate anaerobe bacteria. During and after the reperfusion phase of IRI, luminal oxygen increases substantially^339^ which promotes growth of facultative anaerobic Proteobacteria. While considered the defining characteristic of the IRI‐induced shift in microbiota,^340^ the shift requires several days.^341^, ^342^ Whether microbiome monitoring will become a useful screening tool for IRI remains unclear.

## 7 TREATMENT OF HYPOXIA AND DYSOXIA IN INTESTINAL ISCHAEMIA–REPERFUSION INJURY

### 7.1 Current clinical approaches

Treatment of intestinal hypoxia and dysoxia in IRI generally focuses on physiological mechanisms, regulatory functions and abnormalities at all biological levels. Oxygen supply to the intestine depends on appropriate functioning of convective and diffusive components of the transport system and intestinal mitochondrial function. Currently, intestinal oxygenation is difficult to directly assess at bedside and is best approached by surrogates such as lactate, microcirculatory perfusion and carbon dioxide pressure gradients (Table 4).^343^, ^344^

**TABLE 4:** Available techniques to evaluate the microcirculation and tissue oxygenation at bedside.

Method	Measured variable	Advantages	Limitations and comments
Clinical examination (capillary refill time, mottling score, central‐to‐toe temperature gradient)	Regional peripheral perfusion	Direct evaluation No technological device required Easy and rapid applicability	Qualitative evaluation
Videomicroscopy (2nd and 3rd generation digital videomicroscopes)	Microcirculatory blood flow, vascular density, perfusion heterogeneity, RBC^a^ velocity	Direct evaluation Gold standard method Semi‐quantitative evaluation Bedside monitoring and titration of therapy	Requires specific tools and training
Partial pressure of mucosal carbon dioxide (gastric and sublingual capnometry)	Tissue carbon dioxide	Quantitative evaluation Bedside assessment of flow adequacy	Technical issues interfering with gastric tonometry measurements
Tissue near infra‐red spectroscopy	Oxygen saturation in microvessels (arterioles, capillaries and venules)	Quantitative evaluation Estimates global decreases in tissue perfusion	Large sampling volume comprising arterioles, capillaries, venules Normal or falsely elevated values in context of flow heterogeneity
Oxygen consumption/delivery relationship	If oxygen consumption is limited by perfusion	Identifies potential benefit for increasing convective oxygen transport	Mathematical coupling if oxygen consumption measured by pulmonary artery catheter Indirect calorimetry limited at high fraction of inspired oxygen and time to achieve steady state
Lactate	Marker of anaerobic metabolism	Easy to measure Associated with outcome	Surrogate measurement of tissue oxygenation Can be of non‐anaerobic origin Long decay time
Lactate/pyruvate ratio	Marker of anaerobic metabolism	Confirm anaerobic origin of lactate	Surrogate measurement of tissue oxygenation Cumbersome to measure
SvO_2_ ^b^/ ScvO_2_ ^c^	Balance between oxygen consumption and delivery	Estimates global decreases in tissue perfusion/oxygen supply	Surrogate measurement of tissue oxygenation Requires pulmonary artery or central venous catheter May be normal or elevated when heterogeneity of blood flow is increased ScvO_2_ may not reflect SvO_2_ in conditions of splanchnic hypoperfusion
Pv‐aCO_2_ ^d^	Marker of tissue perfusion	Easy to measure Surrogate of microvascular perfusion assessment	Surrogate measurement of tissue oxygenation Influenced by Haldane effect
∆Pv‐aCO_2_/∆Ca‐vO_2_ ^e^ ratio	Marker of anaerobic metabolism	Surrogate of respiratory quotient Detects anaerobic origin of lactate	Surrogate measurement of tissue oxygenation Influenced by Haldane effect May be influenced by venous H^+^ accumulation Cumbersome calculation
NADH^f^ fluorescence	Marker of anaerobic metabolism	Can be applied on any microscope Does not require dye infusion	Surrogate measurement of tissue oxygenation Not yet available for bedside measurements

Modified from Refs. 343 and 344.

^a^ Red blood cell.

^b^ Venous oxygen saturation.

^c^ Central venous oxygen saturation.

^d^ Venous minus arterial carbon dioxide partial pressure.

^e^ Central venous‐to‐arterial carbon dioxide difference to arterial‐to‐venous oxygen content difference ratio.

^f^ Nicotinamide adenine dinucleotide.

Intestinal ischaemia and reperfusion are often caused or accompanied by hypotension which would normally prompt fluid administration. However, available studies^345^, ^346^, ^347^, ^348^ and pathophysiological rationale suggest that excessive fluid administration can produce excessive intra‐abdominal pressure^349^, ^350^ which can impair intestinal oxygen availability and therefore aggravate IRI. Current evidence is insufficient to recommend specific fluid volume strategies for patients with or at risk for increased intra‐abdominal pressure.

The revised Starling equation indicates that trans‐endothelial fluid movement is related to the plasma‐sub‐glycocalyx colloid osmotic pressure. Consequently, colloids such as albumin may delay trans‐vascular fluid escape.^351^ On the other hand, colloids may increase hydrostatic capillary pressure which can augment fluid filtration. Crystalloid solutions decrease colloid osmotic pressure and increase hydrostatic capillary pressure, theoretically leading to higher fluid filtration than colloids.^351^ As with fluid volume, there is currently no evidence‐based guidance for selecting crystalloid or colloid for IRI.

Abdominal perfusion pressure is estimated as the mean arterial pressure minus the intra‐abdominal pressure which is usually 0–5 mmHg. Elevated intra‐abdominal pressure reduces blood flow to the abdominal viscera including intestines. Clinical^352^, ^353^, ^354^ and experimental^355^, ^356^, ^357^ evidence suggest that a target abdominal perfusion pressure of at least 60 mmHg improves survival after intestinal hypoperfusion.^358^, ^359^, ^360^

Based on the rationale provided by the Starling curves and Guyton theory of cardiac function,^361^ elevated central venous pressure also increases splanchnic venous pressure and decreases intestinal perfusion and capillary exchange capacity. Central venous pressure is normally low, and even below zero when sitting quietly.^362^ When intestinal perfusion is insufficient, a reasonable strategy is to first reduce central venous pressure which will also reduce splanchnic congestion and improve lymphatic flow.^363^ Thereafter, as necessary, arterial pressure can be increased.

It is equally important to consider that the veins of patients with severe hypovolemia are usually maximally constricted. Further activation of alpha‐adrenergic receptors in veins with exogenous pure α‐1 adrenergic agonists thus has little effect on venoconstriction, but does constrict arteries potentially leading to intestinal hypoperfusion and hypoxia.^364^, ^365^, ^366^, ^367^, ^368^, ^369^ Compared with α‐1 adrenergic agonists, norepinephrine might exert an additional benefit by stimulating β‐2 adrenoceptors which may facilitate emptying of the venous system and possibly enhance visceral blood flow. We note though that catecholamines may affect coagulation through pathways other than those involving adrenergic receptors^370^ and inhibit cellular respiration in a dose‐dependent manner,^371^, ^372^ thus aggravating microvascular flow and cellular metabolism.^43^ Reducing endogenous and exogenous adrenergic stimulation (decatecholaminisation) and using a multimodal vasopressor strategy may prevent or reduce the immune‐ and metabolism‐modulating properties of exogenous catecholamines^373^ and ameliorate metabolic stress.^374^

Common treatment strategies based on the correction of macrohaemodynamic variables often do not account for downstream microcirculatory failure. Indeed, relying purely on macrohaemodynamic targets when trying to restore intestinal tissue oxygen delivery may be futile since haemodynamic coherence may be lost. Consequently, classical haemodynamic optimisation may fail to improve microvascular perfusion and even prove deleterious. Therefore, a microcirculation‐guided resuscitation strategy might improve intestinal perfusion and haemodynamic coherence during IRI—recognising that we currently lack reliable ways of evaluating intestinal microcirculation in patients (Table 4). Vasodilators or more sophisticated strategies such as blood purification and antioxidants may also be effective.^375^, ^376^, ^377^ But as with other aspects of fluid management for IRI, there is currently little evidence to suggest that any given approach is preferable. Importantly, macro‐ and micro‐circulatory responsiveness to treatments should be simultaneously assessed to determine whether improved macrohaemodynamics improve intestinal capillary flow.

### 7.2 Experimental therapeutics and potential interventions

Various treatment approaches for post‐IRI hypoxia and dysoxia have been assessed in research settings with mixed results. Oxygen microbubbles and other chemicals that off‐load oxygen can be delivered intra‐luminally.^378^, ^379^ However, there is little evidence of benefit^378^ while hyperoxia might induce vasoconstriction in the microcirculation, thereby worsening perfusion heterogeneity, and decrease endothelial cell viability and proliferation.^380^, ^381^, ^382^, ^383^

The mPTP is a calcium‐dependent, ion non‐selective membrane channel that mediates inner mitochondrial membrane permeability, allowing diffusion of molecules up to 1.5 kDa in size.^384^ Short‐term (reversible) opening of the mPTP contributes to mitochondrial bioenergetics, protection from oxidative damage, enabling the efflux of calcium from the mitochondrial matrix, and cell signaling.^385^ Sustained (irreversible) mPTP opening causes mitochondrial swelling, which ruptures the outer mitochondrial membrane and induces processes leading to cell death. Intestinal IRI induces calcium overload in mitochondria and the production of reactive oxygen species, which causes the mPTP to open.^224^, ^225^, ^226^ As a result, molecules with small molecular weights and H^+^ anions enter the mitochondrial matrix, dissipating the mitochondrial membrane potential, uncoupling the electron transport chain and inhibiting adenosine triphosphate synthesis,^386^ while water seeps into the organelles which causes them to swell and rupture.^387^

Experimental evidence suggests that tight serum glucose control may preserve mitochondrial function and attenuate organ dysfunction independently of organ perfusion.^388^ Metformin may help attenuate mPTP opening, stimulate mitochondrial biogenesis and reduce mitochondrial reactive oxygen species production. However, the drug can also cause metabolic acidosis with hyperlactatemia, vasoplegia and hypoglycemia.^389^, ^390^ Imeglimin inhibits mitochondrial permeability transition, possibly with less toxicity than metformin.^389^, ^391^ Animal studies also suggest that cyclosporine A inhibits the mPTP in different conditions, that is, sepsis and traumatic brain injury. Studies targeting restoration of cytochrome‐*c*‐oxidase (Complex IV) activity and maintenance of mitochondrial inner membrane integrity suggest that antioxidant treatments may preserve intestinal function by maintaining mitochondrial function.^392^, ^393^, ^394^

An intriguing therapeutic approach is ischaemic pre‐ and post‐conditioning which might enhance the HIF cascade and improve resistance to intestinal IRI. Specifically, remote ischaemic preconditioning, achieved through repeated and brief ligation of a limb prior to the induction of intestinal ischaemia, reportedly reduces microscopic damage to the intestine. Reduced damage correlated with reduced concentrations of myeloperoxidase and reduced HIF‐1a expression, supporting the hypothesis that ischaemic preconditioning ameliorates intestinal IRI.^395^, ^396^ Ischaemic preconditioning induces upregulation of miR‐21 through HIF‐1α, inhibits apoptosis and leads to downregulation of the apoptotic mediators PDCD4 and Fas‐L, thereby mitigating intestinal I/R injury.^397^

Ischaemic postconditioning is safe and clinically feasible and may improve the morphology and respiratory function of intestinal mucosal cell mitochondria and increase mitochondrial transmembrane potential.^387^ For example, three cycles of 5‐min ischaemia/5‐min reperfusion induced by a blood pressure cuff placed on the upper limp may attenuate intestinal injury in patients undergoing major non‐cardiac surgery without any potential risk.^398^, ^399^ Experimental evidence suggests that ischaemic postconditioning alleviates intestinal mucosal injury and oxidative stress by regulating mPTP formation to ameliorate intestinal I/R injury.^400^ Additionally, ischaemic postconditioning reduces intestinal injury by inhibiting apoptosis of intestinal mucosal cells.^401^, ^402^

Manipulation of intravascular volume as well as the coagulation profile in the setting of haemorrhage with fresh frozen plasma, modification of microvascular shear stress and use of novel vasoactive agents may be protective against IRI and IRI‐associated hypoxia/dysoxia.^43^, ^403^, ^404^, ^405^ Downstream regulation of the hypoxia cascade by chelation of iron‐catalysed reactive oxygen species has been attempted with the novel iron‐chelator DIBI, with promising results.^406^ Also, melatonin, although having no effect on macrohaemodynamics variables, may exert anti‐inflammatory and anti‐oxidative action and attenuate the shock‐induced decrease of microcirculatory oxygenation.^407^

Other experimental studies suggest that the IRI‐induced vascular permeability is ameliorated by hypothermia during the ischaemia and reperfusion.^408^ Increased permeability following combined ischaemia and hypothermia was observed only when reperfusion was accompanied by rewarming.^408^ These data suggest that hypothermia may be a protective physiologic response to injury and that hypothermic reperfusion may limit capillary endothelial damage and oedema formation, possibly by decreasing the synthesis of chemical mediators and oxygen radicals. Furthermore, hypothermia has been shown to reduce the degradation of adenosine triphosphate to hypoxanthine and the rate of conversion of xanthine dehydrogenase to xanthine oxidase,^409^ thereby protecting against mucosal permeability and intestinal IRI. Finally, dexmedetomidine and other anaesthetics may modulate gene expression, channel activation, transmitter release, inflammatory processes and cell death, thus exerting protective effects in intestinal IRI.^410^, ^411^, ^412^

Experimental studies suggest various intriguing approaches. However, we currently lack convincing clinical evidence to support any particular management strategy.

## 8 CONCLUSIONS AND FUTURE PROSPECTS

**FIGURE 11:**
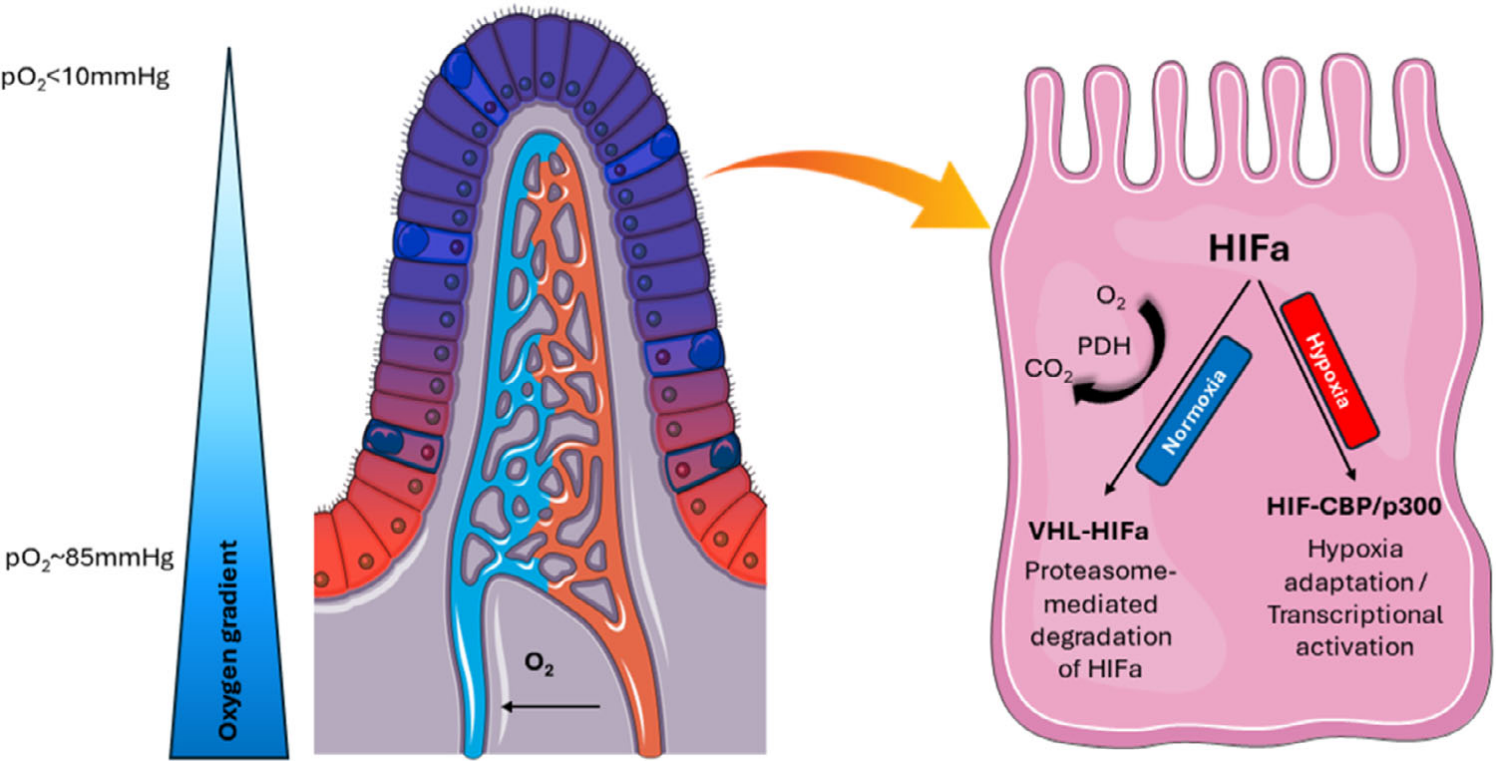
Intestinal oxygen utilisation and cellular adaptation during intestinal ischaemia–reperfusion injury. During ischaemia, mitochondrial oxygen uptake is reduced when cellular oxygen partial pressure decreases to below the threshold required to maintain normal oxidative metabolism. Upon reperfusion, intestinal hypoxia may persist because microcirculatory flow remains impaired and/or because available oxygen is consumed by enzymes, intestinal cells and neutrophils.

The pathology of intestinal IRI is complicated and involves numerous mechanisms organised and integrated into increasing levels of complexity (Figure 11). Hypoxia and dysoxia are important contributors to development and exacerbation of intestinal IRI via various cellular and subcellular mechanisms even though intestinal tissues generally tolerate a degree of hypoxia.

Among the most important protective mechanisms is activation of the HIF system which enhances transcription of genes in various affected cells and triggers biochemical and immunologic pathways to maintain tight junction integrity and mucosal barrier function. However, activation of the HIF system may also augment apoptotic and inflammatory processes, thus aggravating IRI‐induced gut mucosal injury. Downstream effects depend on the onset, duration and suppression of HIF system activation. The poorly characterised consequences of the acute and chronic HIF activation should be major research areas in intestinal IRI, as should the therapeutic potential of targeting HIF activation pathways.

Currently, there are no practical methods of diagnosing intestinal hypoxia and dysoxia during IRI or assessing severity during an episode. Maintaining tissue perfusion and oxygenation presumably prevents worsening of the phenomena and provides time to reverse the underlying aetiology. Better understanding intestinal hypoxia and dysoxia phenotypes and behaviours during IRI will require considerable additional study at various integrative levels. Future research on intestinal oxygen utilisation and cellular adaptation during intestinal IRI should focus on the physiological mechanisms, regulatory functions and abnormal alterations at all biological levels, as well as on their complex interactions and integration.

## AUTHOR CONTRIBUTIONS

A. C. conceptualised the article. P. A. B., G. B., E. L. and A. C. drafted manuscript. T. M. prepared figures. P. A. B., T. M., D. D. C., G. B., E. L., D. I. S., G. G. and A. C. edited and revised manuscript. All authors approved the final version of manuscript.

## CONFLICT OF INTEREST STATEMENT

The authors declare no conflict of interest.

## FUNDING INFORMATION

The authors declare that no funding was received for this review.

## ETHICS STATEMENT

Not applicable.

## Data Availability

Data sharing not applicable to this article as no datasets were generated or analysed during the current study.
